# A DNA Tetrahedron–Delivered miR-17-5p mimic exerts dual anti-inflammatory and Anti-*Pseudomonas aeruginosa* effects in cystic fibrosis

**DOI:** 10.1016/j.ncrna.2026.06.006

**Published:** 2026-07-29

**Authors:** Yunfei Ye, YinHeRichard Sun, Hawraa Shahrour, Irene Oglesby, Yiran Zheng, Catherine M. Greene

**Affiliations:** a Department of Clinical Microbiology, RSCI University of Medicine & Health Sciences, Dublin, Ireland; b Department of Medicine, Royal College of Surgeons in Ireland, Dublin, Ireland; c College of Pharmaceutical Sciences, Soochow University, Suzhou, China

**Keywords:** Cystic fibrosis, *Pseudomonas aeruginosa*, miR.17.P4d_5p, DNA tetrahedron, Antimicrobial resistance, Biofilm formation

## Abstract

Cystic Fibrosis (CF) is a genetic disease characterized by chronic pulmonary inflammation and infection, principally with *Pseudomonas aeruginosa*. *P. aeruginosa* can exhibit resistance to innate immune effectors and antibiotics by expressing virulence factors and forming antibiotic-resistant biofilms, which facilitate chronic infection in immunocompromised hosts. Therefore, new antimicrobials are required. MicroRNAs are mammalian short non-coding RNAs that negatively regulate protein expression. MiR-17-5p, an inhibitor of CXCL8/IL-8, can decrease neutrophil abundance in the CF lung. Here we investigate whether miR-17-5p has *anti*-*P. aeruginosa* properties. The sequences of the full-length miRNA and the miR-17-5p isoform miR.17.P4d_5p were tested for their ability to bind to RNA transcripts encoded by *P. aeruginosa* PAO1 (NC_002516) using the bioinformatics tools RocketmiR and IntaRNA. A miR-17-5p DNA tetrahedron (Td-miR.17.P4d_5p, Size = 118–180 nm) was tested for its delivery into CF bronchial epithelial cells and ability to inhibit IL-8 gene and protein expression, and its effects on *P. aeruginosa* growth, antimicrobial resistance, and biofilm formation. Successful delivery into CFBE41o− and 16HBE14o− cells inhibited IL-8 expression induced by *Pseudomonas*-conditioned medium (PAO1, Mucoid strains 1 and 2 *p* < 0.00001; PA14 *p* < 0.0001). Delivery into PAO1 and PA14 inhibited growth and biofilm formation while significantly enhancing their sensitivity to the antibiotic cefotaxime. Td-miR.17.P4d_5p also significantly inhibited biofilm formation in two clinical mucoid *P. aeruginosa* strains. qPCR and EMSA were used to investigate the mechanism of action of miR.17.P4d_5p. The data showed that miR.17.P4d_5p could inhibit expression of *kynU* (*p* < 0.00001), a tryptophan metabolism pathway molecule related to PAO1 growth, and the biofilm formation-related molecules *pilL*, *fimX*, *ppkA* and *pslA* (all *p* < 0.00001) in *P. aeruginosa* PAO1. The combined *anti*-IL-8 and *anti*-*P. aeruginosa* properties of miR.17.P4d_5p suggest its potential usefulness as a dual anti-infective and anti-inflammatory therapeutic strategy for CF.

## 1 Introduction

Cystic fibrosis (CF) is an autosomal recessive genetic disease whose main pathological feature is abnormal mucus and sweat gland function in multiple organs, but especially the lungs, leading to death in 90% of patients [1]. CF is a genetic disorder marked by chronic lung inflammation and persistent infections, primarily caused by *Pseudomonas aeruginosa*. Despite significant advancements in the health and quality of life of people with CF due to the development of cystic fibrosis transmembrane conductance regulator (CFTR) modulator therapies (such as Trikafta/ETI), which restore CFTR function, infections in the CF lung—especially those involving *P. aeruginosa*—remain a significant challenge [2]. The eradication of *P. aeruginosa* from the CF lung is clinically difficult due to its capacity to form biofilms and develop antimicrobial resistance (AMR) [3].

CFTR genotypes determine the mechanism underpinning the CFTR channel dysfunction in people with CF (pwCF), which ultimately leads to dehydration of the mucosal surface and an increase in mucus that blocks the airways, pancreas, and intestines [4]. At the same time, a large number of neutrophils infiltrate into the lungs and release elastase, causing damage to the lung tissue [5]. Consequently, a routine for most pwCF involves the daily use of inhaled medications designed to decrease the viscosity of mucus and nebulization with hypertonic saline [6]. CFTR modulators are a class of small molecule drugs that can directly affect the CFTR protein and can significantly improve the function of the CFTR channel which transports chloride ions, bicarbonate and glutathione. Notwithstanding the benefits of these therapies, infection of the CF lung with *P. aeruginosa* and other bacteria and the associated inflammation, remain a major problem [7,8]. Consequently, anti-inflammatory therapies have become a crucial component of CF management.

MiRNAs are mammalian non-coding RNA that negatively regulate target protein expression by binding in a sequence dependent manner to miRNA recognition elements in the 3′untranslated regions (3′UTR) of target mRNAs to prevent their translation and/or degrade the mRNA within the RNA-induced silencing complex (RISC) [9]. The expression levels of miRNAs are often significantly altered in pathological conditions, such as lung inflammation and disease. This is particularly relevant in CF.

Our group performed the first microRNA expression profiling study in bronchial brushing samples from pwCF [10]. We also demonstrated that miR-17-5p, which is significantly reduced in CF airway epithelium, can inhibit CF airway epithelial cell expression of CXCL8/IL-8 when delivered via nebulization in biocompatible nanoparticles [11,12]. CXCL8/IL-8 is a potent neutrophil chemoattractant and a validated target of miR-17; thus, its inhibition serves to modulate the highly pro-inflammatory environment of the CF lung by reducing excessive neutrophil recruitment. Collectively, these findings establish miR-17-5p as an important regulator of airway inflammation in CF. If miR-17-5p is also experimentally verified to exert functional activity against *P. aeruginosa*, its well-characterized anti-inflammatory properties could be leveraged in parallel with antibacterial activity to develop a novel dual-function therapeutic strategy for CF.

*P. aeruginosa* is a major opportunistic pathogen in CF that readily forms antibiotic-resistant biofilms, contributing to chronic infection and treatment failure. Consequently, strategies that prevent newly acquired infections or eradicate established bacterial populations remain a critical unmet need, particularly given the intrinsic recalcitrance of biofilms to conventional antibiotics and the growing problem of antimicrobial resistance. Koeppen et al. first demonstrated that extracellular vesicles secreted by human airway epithelial cells deliver miRNA Let-7b-5p to *P. aeruginosa*, thereby reducing the abundance of proteins essential for biofilm formation, and increasing the activity of beta-lactam antibiotics by targeting the beta-lactamase AmpC [13]. We reported a similar phenomenon for a miR-101_3p DNA mimic whereby its delivery via tetrahedron technology to either *Staphylococcus aureus* or *P. aeruginosa* inhibited their exponential phase growth, and led to an increase in the bactericidal activity of cefotaxime against *P. aeruginosa* [14]. These and other recent studies implicate miRNAs and mimics thereof as novel antimicrobials [[15], [16], [17], [18]].

Herein we explore the dual effects of a tetrahedron-tethered miR-17-5p DNA mimic on IL-8 expression in CF bronchial epithelial cells and as an *anti*-*P. aeruginosa* agent.

## 2 Methods

### 2.1 *Pseudomonas aeruginosa* strains

*P. aeruginosa* strains 01 (PAO1, ATCC 156920) and 14 (PA14, a gift from A. Crabbé, Ghent University), *P. aeruginosa* mucoid clinical isolates (PA mucoid strain 1 and PA mucoid strain 2, of non-CF origin from S. O'Donnell, Department of Clinical Microbiology, RCSI) were used in this study.

### 2.2 *Staphylococcus aureus* strains

Methicillin-susceptible *Staphylococcus aureus* (MSSA) strain SH1000 [19], methicillin-resistant *S*. *aureus* (MRSA) strains USA300 LAC and BH1CC [20] were used in this study.

### 2.3 CFBE41o− and 16HBE14o− bronchial epithelial cell lines

The human F508del homozygous CF bronchial epithelial cell line CFBE41o− and the human non-CF bronchial epithelial cell line 16HBE14o− were kindly provided by D. Gruenert (California Pacific Medical Center Research Institute, San Francisco, CA, USA) [21]. CFBE41o− and 16HBE14o− cells were cultured in MEM + GlutaMax (Gibco) with 10% fetal calf serum (FCS; Gibco) and 1% penicillin–streptomycin (Pen-Strep; Gibco), as previously reported [12].

### 2.4 MiRNA-like DNA oligos, Td-miR.17.P4d_5p synthesis

The miR.17.P4d_5p DNA oligos were manufactured by Integrated DNA Technologies Europe (IDT) and annealed with four single-stranded DNA oligonucleotides (S1-S4) to generate Td-miR.17.P4d_5p (Supplementary Table S1) [14,22]. Each was diluted using IDT Duplex Buffer to a 100 μM stock solution, and equal volumes of each were mixed and annealed using a heating block set to 95°C for 5 min and allowed to cool to 4°C overnight to form 20 μM Td-miR.17.P4d_5p oligo complexes. A scrambled sequence of hsa.miR.17.P4d_5p (Sc_ miR.17.P4d_5p) was generated as a random sequence control. The formed DNA oligo-tetrahedron complexes (DNAtd) were stored at 4°C and retained their biological effects for at least 1 month after construction. Modifications were applied for imaging purposes, where a Cy5 dye (excitation 647 nm and emission 665 nm) was added onto the S1 strand and a 6-FAM dye (excitation 498 nm and emission 517 nm) was added onto the miR.17.P4d_5p DNA oligo during manufacture.

Nanosight capturing of Td-miR.17.P4d_5p was performed using a NS300 (Malvern Panalytics) under a ×1000 water dilution from the standard annealing process with camera setups as follows; Blue 488 laser, camera level 10, slider shutter 696, slider gain 55, FPS 25.0, number of frames 1,498, temperature 19.9°C, viscosity 1.003–1.004 cP. An averaged finite track length adjustment concentration/size curve was generated using five separate reads from the sample. TEM was performed using a H7650 Transmission Electron Microscope (Hitachi) with undiluted DNAtds from the standard annealing process with images generated using a higher voltage of 100 kV [14].

### 2.5 Preparation of Td-miR.17.P4d_5p + LP complexes

A defined concentration of Td-miR.17.P4d_5p (e.g., 1 μM) was mixed with Lipofectamine 2000 (LP2000, Invitrogen) at a fixed ratio (0.04 μL LP per μL Td solution). The mixture was incubated at room temperature for 15 min to allow the formation of Td-miR.17.P4d_5p + LP complexes [14,23].

### 2.6 Td-miR.17.P4d_5p delivery detection

Flow cytometry was employed to assess the transfection efficiency of Td–miR-17-P4d-5p labeled with 6-FAM and Cy5 dye in CFBE cells. Briefly, 1 × 10^5^ CFBE cells were seeded per well in 24-well plates and incubated with 1 μM/0.5 μM/0.25 μM/0.125 μM Td–miR-17-P4d-5p–6-FAM & Cy5 at 37°C for 8 h. Following incubation, the culture medium was removed, and the cells were washed twice with 1 × PBS. Cells were then detached using trypsin to generate single-cell suspensions and washed once with FACs buffer (1 mL fetal calf serum added to 49 mL 1 × PBS). Finally, cells were resuspended in FACs buffer to a final volume of 150 μL per sample. The fluorescence intensity of 6-FAM and Cy5 labels in CFBE cells was assessed by detecting the FITC and Cy5 channels, respectively, to determine the uptake efficiency of Td–miR-17-P4d-5p by CFBE cells.

Confocal microscopy was used to visualize delivery of Td-miR.17.P4d_5p-6-FAM into PAO1. Overnight cultures were prepared using Mueller Hinton Broth (MHB), shaken and incubated at 37°C. Bacterial control and Td-miR.17.P4d_5p transformation samples were prepared with 10 mL of MHB with 100 μL of overnight culture. Td-miR.17.P4d_5p were added to a final concentration of 1 μM and samples were shaken and incubated at 37°C for 4 h with LP (0.04 μL LP per μL Td solution) added to the PAO1 group. Samples were shielded from light and gently heat fixed to microscopy slides to prevent fluorescence quenching. The ZEISS LSM 710 microscope system was used to image the bacterial samples. An excitation *λ* at 488 nm was used for 6-FAM label attached to the miR.17.P4d_5p DNA oligo. Images were captured at ×64 and merged with a brightfield image to indicate transformation of Td-miR.17.P4d_5p.

### 2.7 PAO1, SH1000, USA300, and BH1CC growth analysis

In order to measure the effect of Td-miR.17.P4d_5p on PAO1, SH1000, USA300, and BH1CC growth, Td-miR.17.P4d_5p oligo complex ± LP (0.04 μL LP per μL Td solution) was used to determine if transfection reagent is required for effective delivery into *P. aeruginosa* and *Staphylococcus aureus*. Overnight cultures were prepared from CRYOBANK™ bead stocks using MHB, shaken and incubated at 37°C. Four experimental groups were setup as follows: group 1 (non-miR control), group 2 (non-miR control + LP), group 3 (Td-miR.17.P4d_5p), and group 4 (Td-miR.17.P4d_5p + LP). LP and 1 μM Td-miR.17.P4d_5p at were added to 5 × 10^7^ bacterial cells at the start of the experiment (T = 0 h). Culture media, 20 μL, of each group was removed at T = 0, 2, 4, 6, 8, 24 h, plated on Mueller–Hinton agar, incubated at 37°C overnight and CFU/mL was calculated for each condition.

### 2.8 Evaluation of Td-miR-17-P4d-5p–mediated enhancement of cefotaxime bactericidal efficacy by killing assay

Overnight cultures of PAO1 and PA14 were diluted in MHB to achieve an absorption reading of OD_500_ 0.1. Aliquots of 100 μL of the diluted bacterial suspensions were distributed into the following treatment groups: (1) non-miR control, (2) non-miR control + LP, (3) 0.5 μM Td-miR.17.P4d_5p + LP, (4) 1 μM Td-miR.17.P4d_5p + LP, (5) 1.5 μM Td-miR.17.P4d_5p + LP. Each treatment group was co-incubated with four different concentrations of cefotaxime (8, 16, 32 or 64 μg/mL) in a 37°C incubator. 10 μL culture media of each group was removed at T = 4, 6, 8 h, plated on Mueller–Hinton agars, incubated at 37°C overnight and colonies were photographed and counted to evaluate bactericidal effects for each condition.

### 2.9 Assessment of Td-miR-17-P4d-5p–mediated enhancement of cefotaxime by bacterial viability assay

BacTiter-Glo™ Microbial Cell Viability Assay was used to measure PAO1, PA14, PA mucoid strains 1 and 2 viability by luminescence. Overnight cultures were diluted in MHB to achieve an absorption reading of OD_500_ 0.1 and 100 μL of diluted bacteria culture was added to a 96-well plate containing LP (0.04 μL LP per μL Td solution), cefotaxime (16 or 32 μg/mL depending on cefotaxime sensitivity of different *P. aeruginosa* strains) and Td-miR.17.P4d_5p or scrambled oligo complex (1 μM) in MHB (PAO1 and PA14) or RPMI + 10% FCS (PA mucoid strains 1 and 2), as indicated, to a final volume of 200 μL. Next, equal volumes (100 μL) of bacterial culture and luminescence assay solution were added to flat bottom opaque white 96-well plates at T = 0, 2, 4, 6, 8 h and blocked from light until relative luminescence units (RLU) were recorded at each time point using a Tecan Spark multimode microplate reader.

### 2.10 Reverse transcription quantitative polymerase chain reaction (RT-qPCR)

Total bacterial RNA was isolated using the Invitrogen PureLink™ RNA Mini Kit according to the manufacturer's instructions, or total cellular RNA was extracted using the TRIzol reagent (Invitrogen) method. The total amount and purity of the extracted RNA were assessed using a NanoDrop™ 8000 spectrophotometer by measuring absorbance at 260/280 nm and determining RNA concentration (ng/μL). First-strand cDNA synthesis was further performed using the EasyScript First-Strand cDNA Synthesis SuperMix (Transgen). Briefly, 500 ng of total RNA was reverse transcribed in a 20 μL reaction mixture containing 1 μL of anchored oligo (dT)_18_ primer (0.5 μg/μL), 10 μL of 2× ES Reaction Mix, EasyScript RT/RI Enzyme Mix, and RNase-free water to the final volume.

Finally, quantitative PCR was performed on a QuantStudio 5 Real-time PCR system (Applied Biosystems, Thermo) using ChamQ Universal SYBR qPCR Master Mix (Vazyme). Each 20 μL reaction volume contained 10 μL SYBR Master Mix, 2 μL of 5 μM forward and reverse primers (Supplementary Table S2), 1 μL cDNA template, and nuclease-free water. The amplification program consisted of 95°C pre-denaturation for 30 s, followed by 40 cycles, each consisting of 95°C denaturation for 10 s and 60°C annealing/extension for 30 s. Melting curve analysis was performed at 95°C for 15 s, 60°C for 60 s, and 95°C for 15 s to verify primer specificity. Relative gene expression levels were calculated using the 2^−^ΔΔCt method, with proC as the bacterial internal control gene and *β-actin* as the cellular internal control gene.

### 2.11 Cytotoxicity assay

Cytotoxicity was assessed using the CytoTox 96® Non-Radioactive Cytotoxicity Assay Kit (Promega, G1780), a colorimetric assay that quantitatively measures lactate dehydrogenase (LDH), a stable cytosolic enzyme released into the culture medium upon cell lysis. CFBE and HBE cells were seeded at a density of 1 × 10^5^ cells per well in 24-well plates. After cell attachment, cells were subjected to one of the following three treatments: (1) no addition (control/ spontaneous LDH release group); (2) 2.5% Td-miR.17.P4d_5p (0.5 μM); (3) 5% Td-miR.17.P4d_5p (1 μM). Following treatment, cells were incubated at 37°C for 24 h. After incubation, 50 μL of culture supernatant from each well was transferred to a fresh, flat-bottom 96-well plate. Subsequently, 50 μL of CytoTox 96® Reagent was added to each well, and the plate was protected from light and incubated at room temperature for 30 min. The reaction was terminated by adding 50 μL of stop solution to each well, after which the plate was vented by puncturing each well with a syringe needle. Absorbance was measured at 492 nm within 1 h using a microplate reader. Wells containing culture medium alone were used as background controls. Maximum LDH release was determined by lysing cells from each treatment group with 10× lysis buffer according to the manufacturer's instructions. The average absorbance value of the culture medium wells was subtracted from all experimental and control wells to obtain net LDH release values. These corrected values were then used to calculate the percentage of cytotoxicity according to the following formula:

$$\%\;Cytotoxicity=\frac{Treated\;Sample\;Net\;LDH\;Release-Spontaneous\;Net\;LDH\;Release}{Maximum\;Net\;LDH\;Release-Spontaneous\;Net\;LDH\;Release}$$

Treated Sample Net LDH Release: LDH release signal (OD_492_) from cells exposed to the treatments.

Spontaneous Net LDH Release: LDH release signal (OD_492_) from untreated control cells (baseline cell death).

Maximum Net LDH Release: LDH release signal (OD_492_) from all treated cell lysis, representing 100% death.

### 2.12 IL-8 ELISA

Human IL-8 protein levels in cell culture supernatants were quantified using the Human IL-8 ELISA MAX™ Deluxe Set (BioLegend, Cat. No. 431504) according to the manufacturer's instructions. CFBE and HBE cells were seeded in 48-well tissue culture plates at a density of 50,000 cells per well and cultured under the indicated experimental conditions for 24 h. Following treatment, culture supernatants were collected and centrifuged to remove cellular debris, then stored at −80°C until analysis. Repeated freeze–thaw cycles were avoided.

High-binding 96-well ELISA plates were coated overnight at 4°C with human IL-8 capture antibody diluted in 1× Coating Buffer A. Plates were washed four times with wash buffer (PBS containing 0.05% Tween-20) and blocked with 1× Assay Diluent A for 1 h at room temperature with shaking. After washing, 100 μL of standards or samples were added per well. IL-8 standards were prepared in 1× Assay Diluent A, starting at 1000 pg/mL followed by serial two-fold dilutions. Samples were assayed without dilution unless otherwise indicated. Plates were incubated for 2 h at room temperature with shaking and then washed. Biotinylated detection antibody was added and incubated for 1 h, followed by avidin–horseradish peroxidase for 30 min at room temperature. After a final series of washes, TMB substrate was added and the reaction was allowed to develop for 15 min in the dark before stopping with stop solution. Absorbance was measured at 450 nm using a microplate reader. IL-8 concentrations were calculated from a standard curve generated using a four-parameter logistic regression.

### 2.13 ssDNA hybridisation electrophoretic mobility shift assay (EMSA)

Electrophoretic mobility shift assays (EMSA) were performed to evaluate the direct interaction between miR.17.P4d_5p and the predicted *P. aeruginosa* target RNAs. DNA probes corresponding to the IntaRNA-predicted binding region were synthesized by IDT, including a wild-type (WT) probe and a mutant (Mut) probe containing three consecutive nucleotide substitutions (C/G to A) within the predicted miR.17.P4d_5p binding site. Each DNA probe consisted of the target sequence flanked by approximately 20 native nucleotides on either side, resulting in a final length of approximately 40 nt (Table 1).

**Table 1:** DNA probes used in EMSA.

Gene	DNA probe type	Binging area	Sequence	Length (nt)	Energy
pslA	Wild type	**445**–**460**	AAACGCGGCATG**TACCTGCAACGGACGG**TGATCCTCGGCT	40	−12.77
	Mutation	**433**–**472 (3 C/G→A)**	AAACGCGGCATG**TACCTGCAAAAAACGG**TGATCCTCGGCT	40	−7.42
ppkA	Wild type	**259**–**275**	ATGGCCATGGAA**TACCTGCCCAACGGCAC**GCTCAAGGAGCG	41	−12.23
	Mutation	**247**–**287 (3 C/G→A)**	ATGGCCATGGAA**TACCTGCCCAAAAACAC**GCTCAAGGAGCG	41	−3.62
ampR	Wild type	**30**–**37**	GCCGCTGAACGC**CCTGCGCG**CCTTCGAAGCTT	32	−6.89
	Mutation	**18**–**49 (3 C/G→A)**	GCCGCTGAACGC**CCTAAACG**CCTTCGAAGCTT	32	Not-predicted
pilI	Wild type	**171**–**186**	GGTCGGCGAAGT**CCTGCACGAACCGCGC**TACACCCAGCTG	40	−17.01
	Mutation	**159**–**198 (3 C/G→A)**	GGTCGGCGAAGT**CCTGCACGAAAAACGC**TACACCCAGCTG	40	−13.34
kynU	Wild type	**537**–**555**	CTACAAGACCGG**CTACCTGCACGACATGCGC**GAGGTCACCCGC	43	−19.39
	Mutation	**525**–**567 (3 C/G→A)**	CTACAAGACCGG**CTACCTGCACGACATAAAC**GAGGTCACCCGC	43	−17.16
fimX	Wild type	**1921**–**1934**	CTGATCGCCGAA**CTGCACGAACAGCA**GAAGCTCAGCAT	38	−17.07
	Mutation	**1909**–**1946 (3 C/G→A)**	CTGATCGCCGAA**CAAAACGAACAGCA**GAAGCTCAGCAT	38	−11.23
miR.17.P4d_5p DNA Oligo (miR 17)			CAAAGTGCTGTTCGTGCAGGTAG	23	

Note: Lower binding energy indicates higher potential binding affinity.

For binding reactions, 2 pmol of miR.17.P4d_5p DNA oligonucleotide and 2 pmol of DNA probe were incubated in 1× binding buffer (10 mM HEPES, pH 7.5, 33.3 mM potassium acetate, and 5% glycerol) in a final volume of 20 μL. Samples were heated at 95°C for 3 min and then allowed to cool gradually to room temperature for 60 min to facilitate hybridisation.

Reaction mixtures were resolved on 1.5 mm 10% native TBE gels (29:1 acrylamide:bis-acrylamide) prepared in 0.5× TBE buffer (Fisher Bioreagents, BP1333-1). Prior to electrophoresis, 2 μL of 10× Blue Juice Loading Buffer (Invitrogen) was added to each reaction mixture. And a 100bp DNA ladder (Invitrogen) was included on each gel as a molecular size reference. Electrophoresis was performed in pre-chilled 0.5× TBE running buffer at 90 V for 60 min at 4°C. Following electrophoresis, gels were stained with 1× SYBR DNA Stain (Invitrogen, S33102) for 35 min at room temperature with gentle agitation and visualized using an Amersham Imager 600 fluorescence imaging system (Blue, 460 nm excitation, Cy2 filter).

The following samples were analyzed: miR.17.P4d_5p DNA oligo alone (4 pmol), WT probe alone (4 pmol), Mut probe alone (4 pmol), miR.17.P4d_5p DNA oligo plus WT probe (2 + 2 pmol), miR.17.P4d_5p DNA oligo plus Mut probe (2 + 2 pmol), and 1× loading buffer alone as a loading control. Binding specificity was assessed by comparing mobility shifts observed with different groups.

### 2.14 Statistical analysis

Statistical analyses and graph generation were performed using GraphPad Prism version 10.4.1. Statistical analyses were performed using unpaired Student's *t* tests, one-way ANOVA, or two-way ANOVA, as appropriate for the experimental design and data type. Data are presented as the mean ± standard error of the mean (SEM) from three independent experiments. *P* < 0.01 was considered to indicate statistical significance. Statistical significance is denoted as follows: not displayed, not significant (*p* > 0.01); *p < 0.01; **p < 0.001; ***p < 0.0001; ****p < 0.00001.

## 3 Results

### 3.1 Selection of miR.17.P4d_5p for *anti*-*P. aeruginosa* activity

In order to investigate whether miR-17-5p has potential *anti*-*P. aeruginosa* activity and may affect biofilm formation, we used RocketmiR to test all nine different isoforms of miR-17-5p [24]. We selected miR.17.P4d_5p for further study because it had the lowest median energy score and was predicted to have the best inhibitory effect on *P. aeruginosa* **(**Supplementary Table S3**)**. This provided a strong rationale for constructing a miR.17.P4d_5p DNA oligonucleotide tetrahedron.

### 3.2 Td-miR.17.P4d_5p was successfully synthesized

**Fig. 1:**
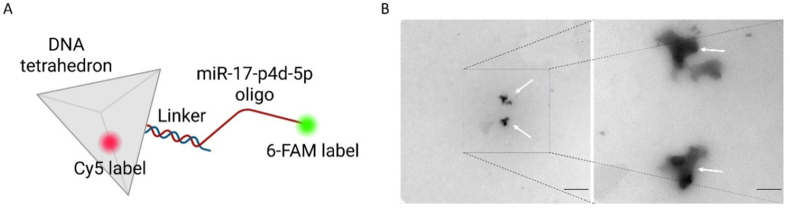
Td-miR.17.P4d_5p structure. (A) Schematic illustration of the Td-miR.17.P4d_5p structure. The DNA oligonucleotides of Td and Hsa.miR.17.P4d_5p achieve antisense complementary binding through a short linker. Td is labeled with Cy5 at the S1 sequence, and the miR.17.P4d_5p DNA oligonucleotide is labeled with 6-FAM at its tail to enable fluorescence-based localization tracking. **(B)** The two-dimensional TEM image shows that Td-miR.17.P4d_5p is triangular in shape, indicating that the Td-miR.17.P4d_5p is a tetrahedral structure. The left panel depicts a 40,000× magnification view of Td-miR.17.P4d_5p, the scale bar is 500 nm. The right panel shows a single Td-miR.17.P4d_5p at 200,000× magnification, the scale bar is 100 nm.

The design and synthesis of Td-miR.17.P4d_5p were based on the S1-S4 sequences published by Goodman et al., in 2005, and utilized a 10 bp linker developed by Thai et al. **(**Supplementary Table S1**)** [22,25]. This linker was added to the S4 sequence and attached via complementary binding to the linker carried by the miR.17.P4d_5p DNA oligo. To facilitate the detection of Td-miR.17.P4d_5p delivery, we added a Cy5 fluorescent label on the S1 sequence and a 6-FAM label on the miR.17.P4d_5p DNA oligo (Fig. 1A). Unlabelled Td-miR.17.P4d_5p was used in non-delivery experiments.

Transmission electron microscopy (TEM) images of Td-miR.17.P4d_5p, magnified at × 40,000 and × 200,000 in two fields of view (Fig. 1B), clearly show the successful assembly of the different oligonucleotide chains into a tetrahedral shape. The Nanosight trace of Td-miR.17.P4d_5p showed a main distribution peak at 118–180 nm, indicating the characteristic size of the Td particles and a concentration of approximately 5 particles per μL (Supplementary Fig. S1). Collectively, these results confirm that Td-miR.17.P4d_5p was successfully synthesized as a tetrahedral nanoscale product.

### 3.3 Td-miR.17.P4d_5p effectively inhibits *P. aeruginosa*–induced IL-8 expression in CFBE and HBE cells

**Fig. 2:**
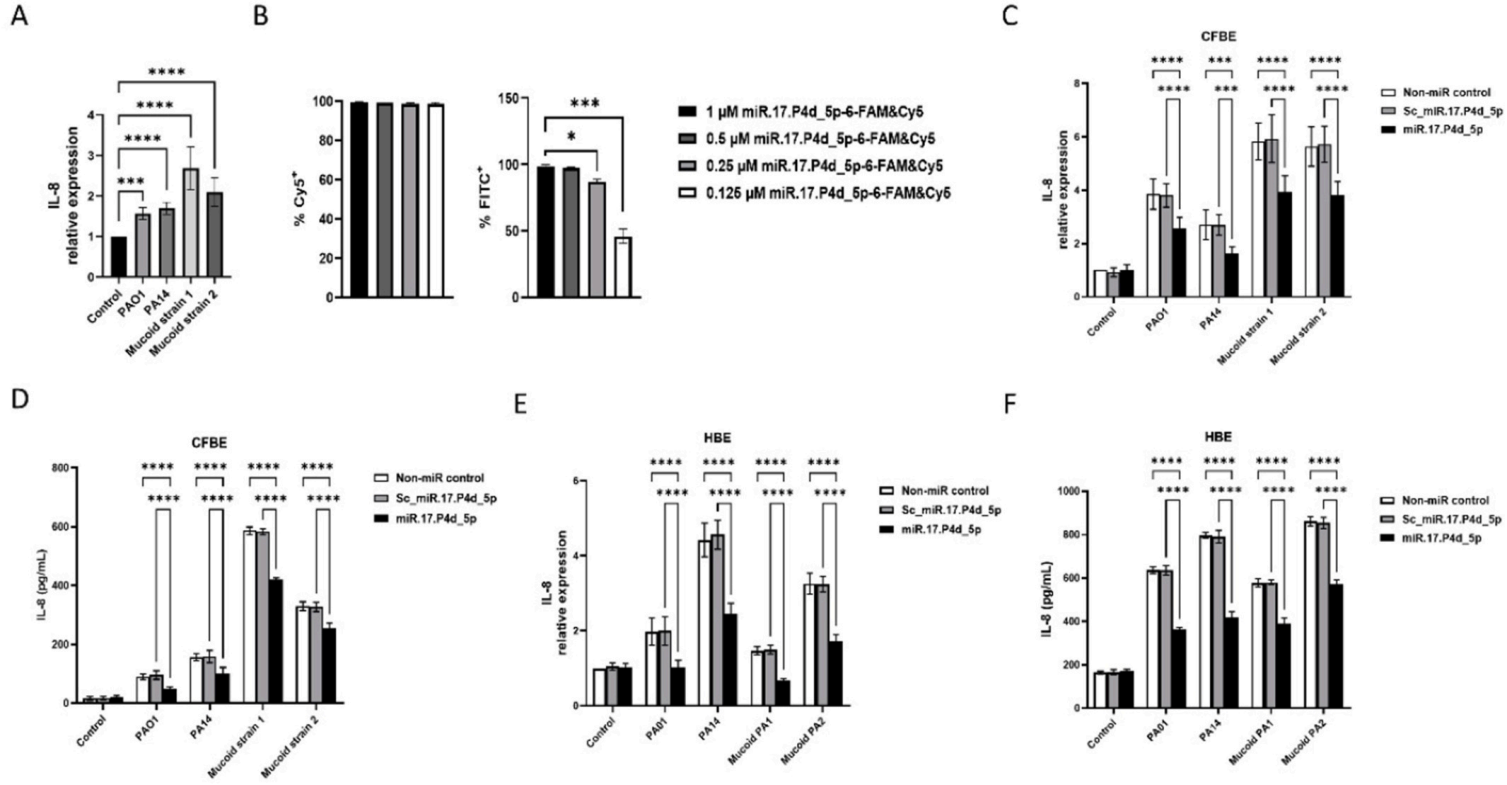
Td-miR.17.P4d_5p inhibits *P. aeruginosa*–induced IL-8 expression in CFBE and HBE cells. **(A)** CFBE cells were treated for 24 h with 10% (v/v) culture filtrates derived from 72-h cultures of four *P. aeruginosa* strains (PAO1, PA14, Mucoid strain 1, and Mucoid strain 2). The Control group was an equal volume of MHB-treated group. Total RNA was extracted from CFBE cells at 24 h and used in qPCR reactions with *IL-8* and housekeeping gene *β-actin* gene-specific primers. Analysis was performed using multiple comparison one-way ANOVA where results were significantly different when comparing treated group to control group with ****p < 0.00001, ***p < 0.0001, not significant (Not displayed, *p* > 0.01), n = 3 biological replicates. **(B)** Cellular uptake of Td-miR.17.P4d_5p labeled with 6-FAM and Cy5 was quantified by flow cytometry across different concentrations. Analysis was performed using multiple comparison one-way ANOVA. ****p < 0.00001, ***p < 0.0001, *p < 0.01, not significant (Not displayed, *p* > 0.01), n = 3 biological replicates. **(C, E)** qPCR analysis of *IL-*8 mRNA expression in CFBE **(C)** and HBE **(E)** cells from different treatment groups after 8 h stimulation with 10% (v/v) culture filtrates from four *P. aeruginosa* strains. IL-8 levels were normalized to *β-actin*. Analysis was performed using multiple comparison two-way ANOVA where results were significantly different when comparing treated group to control group with ****p < 0.00001, ***p < 0.0001, not significant (Not displayed, *p* > 0.01), n = 3 biological replicates. **(D, F)** ELISA analysis of IL-8 protein secretion in **(D)** CFBE and **(F)** HBE cells from different treatment groups after 24 h stimulation with 10% (v/v) culture filtrates from four *P. aeruginosa* strains. Analysis was performed using multiple comparison two-way ANOVA where results were significantly different when comparing treated group to control group with ****p < 0.00001, not significant (Not displayed, *p* > 0.01), n = 3 biological replicates.

Previously we demonstrated that overexpression of miR-17 or miR-17 DNA mimics in cystic fibrosis airway epithelial cells reduced interleukin-8 (IL-8) production [11,12]. To determine whether Td-miR.17.P4d_5p can similarly suppress IL-8 expression, CFBE cells were first treated for 24 h with 10% (v/v) *Pseudomonas*-conditioned medium (PCM) derived from 72-h cultures of four distinct *P. aeruginosa* strains (PAO1, PA14, Mucoid strain 1, and Mucoid strain 2). The qPCR results showed that exposure to all four PCMs resulted in a significant upregulation of IL-8 expression in CFBE cells (Fig. 2A).

Cytotoxicity was assessed using a colorimetric lactate dehydrogenase assay indicating that treatment with 1 μM Td-miR.17.P4d_5p resulted in less than 2% cytotoxicity, meeting the criteria for cell-based experiments **(**Supplementary Fig. S2**)**. Flow cytometry showed that Td-miR.17.P4d_5p was efficiently internalized by CFBE cells over 0.125 to 1 μM, with consistently high Cy5 positivity. In contrast, FITC signals decreased in a concentration-dependent manner, indicating reduced miR.17.P4d_5p retention at lower doses (Fig. 2B). Accordingly, 1 μM was selected as the working concentration for subsequent experiments.

The qPCR analysis revealed that following 8 h stimulation with 10% (v/v) PCM from four distinct *P. aeruginosa* strains, Td-miR.17.P4d_5p significantly reduced IL-8 mRNA expression in CFBE cells compared with both the non-miR control and the Td-scrambled sequence control (Fig. 2C). ELISA demonstrated a significant reduction in PCM-induced IL-8 protein secretion upon Td-miR.17.P4d_5p treatment (Fig. 2D). Consistent results were observed in HBE cells, where Td-miR.17.P4d_5p significantly reduced PCM-induced IL-8 mRNA expression by qPCR (Fig. 2E) and decreased IL-8 protein secretion as measured by ELISA (Fig. 2F), compared with both the non-miR and Td-scrambled sequence controls.

Collectively, these results indicate that Td-miR.17.P4d_5p effectively suppresses *P. aeruginosa*–induced IL-8 expression in CFBE and HBE cells.

### 3.4 Td-miR.17.P4d_5p significantly inhibits the growth rate of PAO1

**Fig. 3:**
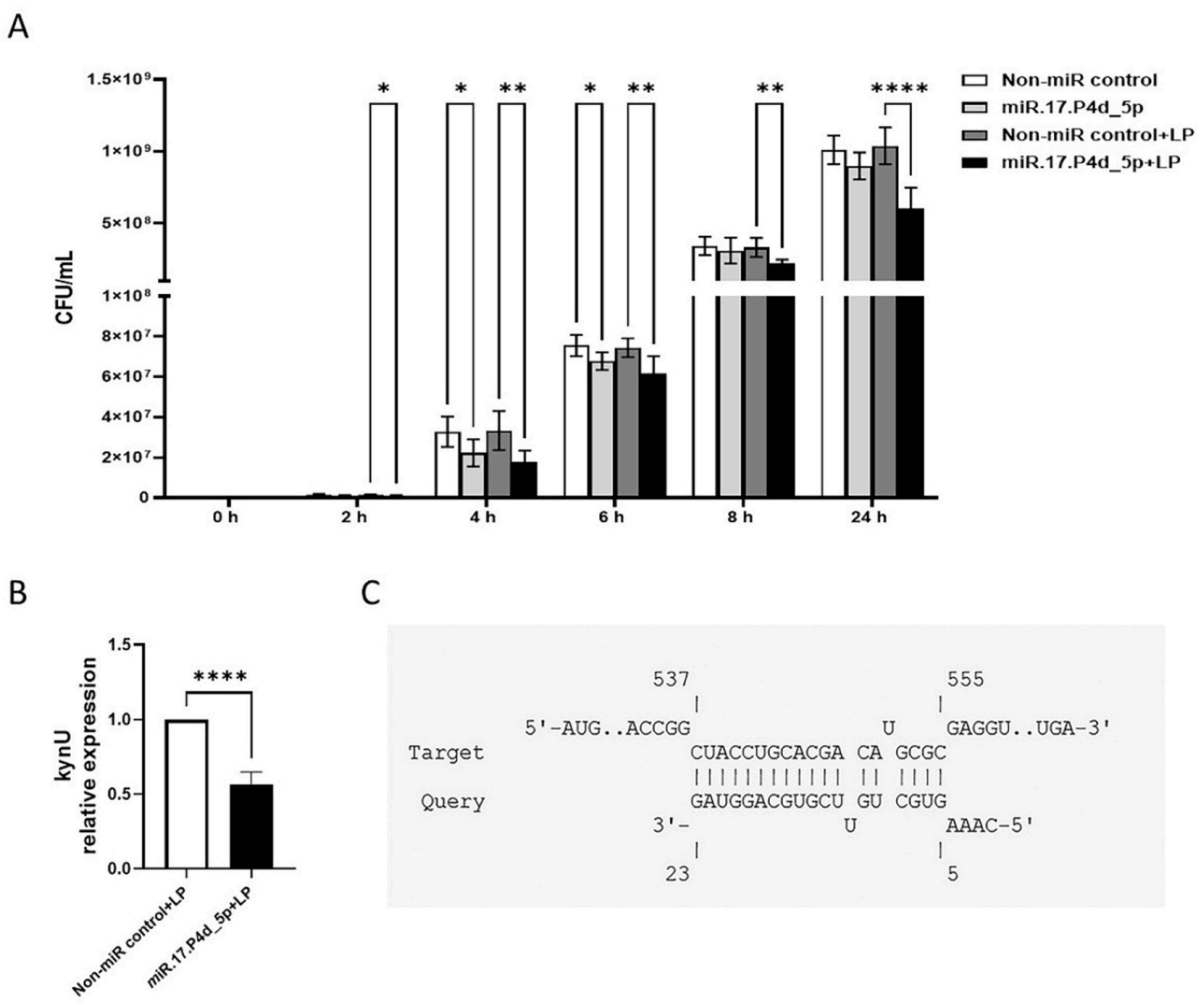
Td-miR.17.P4d_5p inhibits PAO1 growth via targeting *kynU*. **(A)** PAO1 was grown in MHB with and without LP and Td-miR.17.P4d_5p for 24 h and plated at T = 0, 2, 4, 6, 8, and 24 h 1 μM Td-miR.17.P4d_5p −/+ LP were added to 5 × 10^7^ bacterial cells at T = 0 h. The number of viable cells was detected at different time points and presented as CFU/mL −/+ standard deviation. Analysis was performed using multiple comparison two-way ANOVA where results were significantly different when comparing treated group to control group with ****p < 0.00001, **p < 0.001, *p < 0.01, not significant (Not displayed, *p* > 0.01), n = 3 biological replicates. **(B)** qPCR analysis of *kynU* expression in PAO1 at 24 h in the Non-miR control + LP and Td-miR.17.P4d_5p + LP groups, with *kynU* levels normalized to the housekeeping gene *proC*. Analysis was performed using a Student's *t*-test where results were significantly different when comparing treated group to control group with ****p < 0.00001, n = 3 biological replicates. **(C)** IntaRNA visualization of base-pairing interactions between miR.17.P4d_5p and *kynU* RNA.

Next, we investigated whether Td-miR.17.P4d_5p exhibits direct anti–*P. aeruginosa* effects. To assess its effect on bacterial growth, PAO1 cultures were subjected to four treatment conditions, and PAO1 viability was quantified as CFU/mL over time. Results showed that Td-miR.17.P4d_5p significantly reduced PAO1 CFU/mL at 4 h and 6 h regardless of the presence of lipofectamine (LP, a transfection reagent), whereas at 2 h, 8 h, and 24 h, a significant reduction was observed only when LP was present (Fig. 3A). These results indicate that Td-miR.17.P4d_5p can penetrate the PAO1 cell wall and somehow inhibit bacterial growth, and that LP further enhances its uptake and antibacterial activity. Fluorescence microscopy supported this observation, with significant 6-FAM signals detected in PAO1 at 4 h, indicating efficient penetration of the PAO1 cell wall by the Td-miR.17.P4d_5p–6-FAM + LP complex (Supplementary Fig. S3).

To further evaluate the bacterial selectivity of Td-miR.17.P4d_5p, the same growth assay was performed in three *Staphylococcus aureus* strains (SH1000, USA300, and BH1CC). Similar to the observations in *P. aeruginosa*, Td-miR.17.P4d_5p significantly reduced CFU/mL in all three *S. aureus* strains at 6 h and 8 h, regardless of the presence of LP (Supplementary Fig. S4). In addition, growth inhibition was detected at earlier time points (2 h or 4 h) in SH1000 and BH1CC, indicating strain-dependent differences in the onset of the antibacterial response (Supplementary Fig. S4). Collectively, these findings demonstrate that the growth-inhibitory effect of Td-miR.17.P4d_5p is not restricted to *P. aeruginosa* but can also be observed in genetically distinct *S. aureus* strains.

*In silico* target prediction identified a number of binding sites for miR.17.P4d_5p within each of the listed targets (Supplementary Table S4), we focussed on the sites with the lowest binding energy i.e. highest predicted binding affinity. Based on these predictions we further examined the effect of Td-miR.17.P4d_5p in combination with LP on *kynU* expression in PAO1 using qPCR (Fig. 3B). *KynU* encodes kynureninase, a key enzyme in the tryptophan–kynurenine pathway that contributes to bacterial metabolic fitness, quorum sensing, and virulence-associated survival [26]. Td-miR.17.P4d_5p + LP treatment markedly suppressed *kynU* expression, providing mechanistic insight into its growth-inhibitory effect on PAO1. We also used IntaRNA to visualize the base pairing binding between miR.17.P4d_5p and the *kynU* RNA (Fig. 3C) [27].

### 3.5 The beta-lactam antibiotic cefotaxime exerts a stronger antibacterial effect when combined with Td-miR.17.P4d_5p

*P. aeruginosa* is intrinsically resistant to antibiotics because of the particularly low permeability of its outer membrane and the presence of drug efflux pumps, porins, and beta-lactamases [28]. Gentamicin (an aminoglycoside that inhibits protein synthesis) and ciprofloxacin (a fluoroquinolone that interferes with DNA replication) are sometimes used to treat *P. aeruginosa* infections, especially in patients with CF; whereas amoxicillin, oxacillin, and cefotaxime, all of which are beta-lactam antibiotics, are generally not indicated in patients with CF due to the natural resistance of *P. aeruginosa* [29]. Previously we calculated the MIC and MBC of cefotaxime against the *P. aeruginosa* strains utilized here [14]. Among the antibiotics tested, the third-generation cephalosporin cefotaxime exhibited suitable and effective antibacterial activity against both *P. aeruginosa* PAO1 and PA14. Cefotaxime displayed a minimum inhibitory concentration (MIC) of 16 μg/mL and a minimum bactericidal concentration (MBC) of 32 μg/mL against PAO1, compared with an MIC of 32 μg/mL and an MBC of 64 μg/mL against PA14. Cefotaxime was therefore selected to evaluate the potential beta-lactam antibiotic–enhancing effect of Td-miR.17.P4d_5p.

**Fig. 4:**
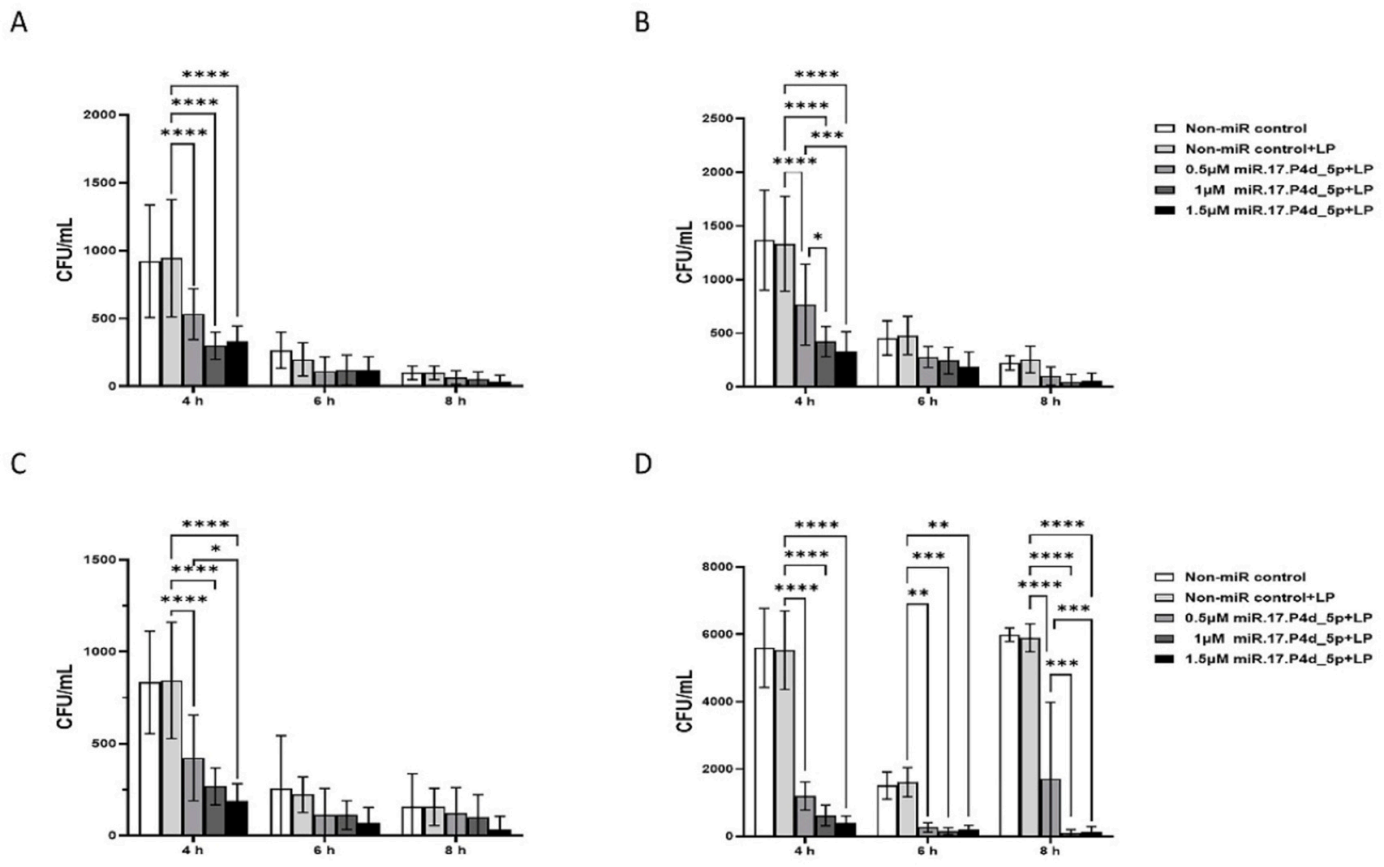
The beta-lactam antibiotic Cefotaxime exerts a stronger antibacterial effect when combined with Td-miR.17.P4d_5p. PAO1 **(A, B)** or PA14 **(C, D)** with an initial concentration of 5 × 10^5^ CFU/mL were left untreated or treated with Td-miR.17.P4d_5p (0.5, 1, or 1.5 μM) −/+ LP (0.04 μL LP per μL Td solution) and different concentrations of cefotaxime **(A**: 64 μg/mL; **B, C**: 32 μg/mL; **D**: 16 μg/mL**)** at 37°C and 150 rpm for 8 h. CFU were determined at 4, 6, and 8 h by plating serial aliquots on MHB agars. Data are presented as CFU/mL histograms. Analysis was performed using multiple comparison two-way ANOVA where results were significantly different when comparing treated group to control group with ****p < 0.00001, ***p < 0.0001, **p < 0.001, *p < 0.01, not significant (Not displayed, *p* > 0.01), n = 3 biological replicates.

PAO1 and PA14 were treated with varying concentrations of Td-miR.17.P4d_5p in combination with a fixed concentration of cefotaxime, and bacterial survival was quantified by CFU/mL. Killing assays revealed no significant differences between the non-miR control and non-miR control + LP groups under all conditions. In contrast, after 4 h of treatment, all concentrations of Td-miR.17.P4d_5p + LP significantly enhanced cefotaxime-mediated killing of PAO1 compared with the non-miR control + LP group. At 32 μg/mL cefotaxime, the 1 μM Td-miR.17.P4d_5p + LP group exhibited greater bactericidal activity than the 0.5 μM group, with no additional benefit observed at the 1.5 μM group (Fig. 4A).

Similarly, in PA14, Td-miR.17.P4d_5p + LP significantly potentiated cefotaxime-induced killing at all tested concentrations after 4 h. Under 16 μg/mL cefotaxime treatment, this enhanced antibacterial effect persisted at 6 h and 8 h. Notably, at 8 h, the 1 μM Td-miR.17.P4d_5p + LP group showed significantly greater killing than the 0.5 μM group, while no difference was observed compared with the 1.5 μM group (Fig. 4B).

MIC and MBC values of cefotaxime in the presence or absence of 1 μM Td-miR.17.P4d_5p were also determined here. The results showed that Td-miR.17.P4d_5p did not alter the conventional MIC and MBC values for cefotaxime (PAO1: 16/32 μg/mL; PA14: 32/64 μg/mL). However, it significantly enhanced the antibacterial activity of cefotaxime against *P. aeruginosa*. This was evidenced by reduced OD_600_ values under sub-MIC conditions (PAO1: 8 μg/mL; PA14: 16 μg/mL) (Supplementary Fig. S5). Collectively, these findings indicate that Td-miR.17.P4d_5p enhances the bacteriostatic activity of cefotaxime against *P. aeruginosa* at sub-MIC concentrations.

Collectively, these findings demonstrate that Td-miR.17.P4d_5p markedly enhances the antibacterial efficacy of the beta-lactam antibiotic cefotaxime against *P. aeruginosa*. Based on its optimal balance of efficacy and consistency, 1 μM Td-miR.17.P4d_5p was selected as the working concentration for subsequent experiments.

### 3.6 Td-miR.17.P4d_5p enhances the bactericidal activity of cefotaxime against *P. aeruginosa* by downregulating the *ampR–ampC* β-lactam resistance axis

**Fig. 5:**
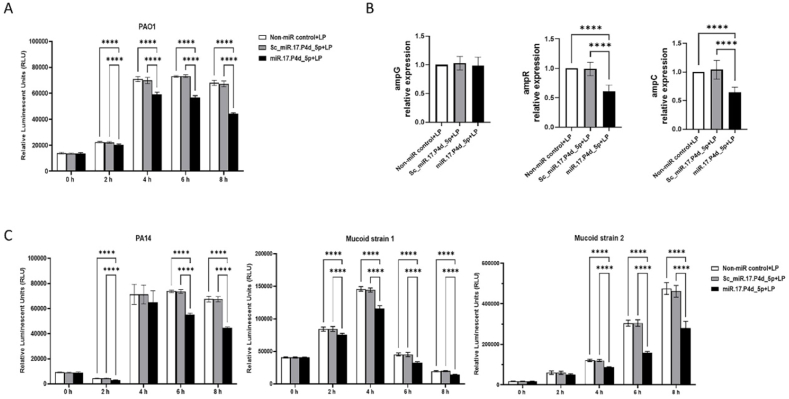
Td-miR.17.P4d_5p enhances the bactericidal activity of cefotaxime against *P. aeruginosa* by downregulating the *ampR–ampC* β-lactam resistance axis. PAO1 **(A)**, or PA14, mucoid strain 1, or mucoid strain 2 **(C)** (5 × 10^5^ CFU/mL) was incubated with LP and either Td-miR.17.P4d_5p (1 μM) or a scrambled control (1 μM) in the presence of cefotaxime at 37°C with shaking (150 rpm) in MHB or RPMI medium supplemented with 10% FBS. Bacterial viability at 0, 2, 4, 6, and 8 h was assessed using the BacTiter-Glo™ Microbial Cell Viability Assay, and RLU was recorded. Analysis was performed using multiple comparison two-way ANOVA where results were significantly different when comparing treated group to control group with ****p < 0.00001, not significant (Not displayed, *p* > 0.01), n = 3 biological replicates. **(B)** Total RNA was extracted from PAO1 at 24 h and used in qPCR reactions with *ampG*, *ampR*, *ampC*, and housekeeping gene *proC* gene-specific primers. Analysis was performed using multiple comparison one-way ANOVA where results were significantly different when comparing treated group to control group with ****p < 0.00001, not significant (Not displayed, *p* > 0.01), n = 3 biological replicates.

The BacTiter-Glo™ microbial cell viability assay is a luminescence-based method that quantifies intracellular ATP to assess microbial viability and is widely used to evaluate the efficacy of antibiotics and antimicrobial agents due to its high sensitivity and accuracy [30]. Using this assay, we further examined the effect of Td-miR.17.P4d_5p on the PAO1-killing activity of 32 μg/mL cefotaxime. Compared with the non-miR control + LP or scrambled sequence control (Sc_miR.17.P4d_5p) + LP, Td-miR.17.P4d_5p (miR.17.P4d_5p) + LP significantly reduced relative luminescence units (RLU) from 2 to 8 h, indicating enhanced bacterial killing (Fig. 5A).

To elucidate the underlying mechanism, we performed qPCR validation of potential target genes *ampG* and *ampR* within the beta-lactam resistance pathway as these were predicted by *in silico* analysis (Supplementary Table S4). In PAO1, AmpG transports peptidoglycan degradation products across the inner membrane, thereby activating the transcriptional regulator AmpR, which subsequently induces expression of the beta-lactamase AmpC and confers resistance to beta-lactam antibiotics such as cefotaxime [[31], [32], [33]]. Given that AmpR is an upstream transcriptional regulator of the beta-lactamase AmpC, we also included *ampC* in the qPCR analysis. Results revealed that miR.17.P4d_5p + LP significantly downregulated both *ampR* and *ampC* expression, while no significant effect was observed on *ampG*, compared with both control groups (Fig. 5B). This demonstrates that although *ampG* was a predicted target of miR.17.P4d_5p the prediction did not hold true, unlike for *ampR*. Expression of *ampC* was indirectly targetted by miR.17.P4d_5p due to inhibition of *ampR* which is a transcription factor essential for its expression.

To determine whether the cefotaxime-enhancing effect of Td-miR.17.P4d_5p observed in PAO1 extends to other *P. aeruginosa* strains, we evaluated its activity against PA14 and two clinically isolated mucoid strains (mucoid strains 1 and 2). In order to better mimic the in vivo clinical environment, assays for the mucoid strains 1 and 2 were performed in RPMI medium supplemented with 10% FBS instead of standard MHB. Cefotaxime exhibited MIC/MBC values against mucoid strains 1 and 2, as previously reported [14]. In PA14, Td-miR.17.P4d_5p treatment resulted in a significant reduction in viability at 2, 6, and 8 h, although no significant change was observed at 4 h. In mucoid strain 1, the cefotaxime-enhancing pattern of Td-miR.17.P4d_5p was similar to that observed in PAO1. Mucoid strain 2, which exhibited greater tolerance to cefotaxime, showed slower growth kinetics and higher RLU values; nevertheless, Td-miR.17.P4d_5p remained effective, albeit with a delayed onset of activity (Fig. 5C).

In summary, Td-miR.17.P4d_5p enhances the bactericidal activity of cefotaxime against both laboratory and clinical *P. aeruginosa* strains by downregulating the *ampR*–*ampC* beta-lactam resistance axis, thereby potentiating antibiotic efficacy across strains with varying levels of drug tolerance.

### 3.7 Effect of Td-miR.17.P4d_5p on biofilm formation and virulence gene expression in *P. aeruginosa*

*P. aeruginosa* readily forms biofilms in the lungs of patients with CF, which is a major contributor to chronic infection and treatment failure. Biofilm formation markedly enhances bacterial tolerance to antibiotics and host immune defenses, promotes mucoid conversion and persistent colonization, and ultimately exacerbates airway inflammation and progressive loss of lung function [[34], [35], [36], [37]]. Accordingly, we investigated the effect of Td-miR.17.P4d_5p on PAO1 biofilm formation.

**Fig. 6:**
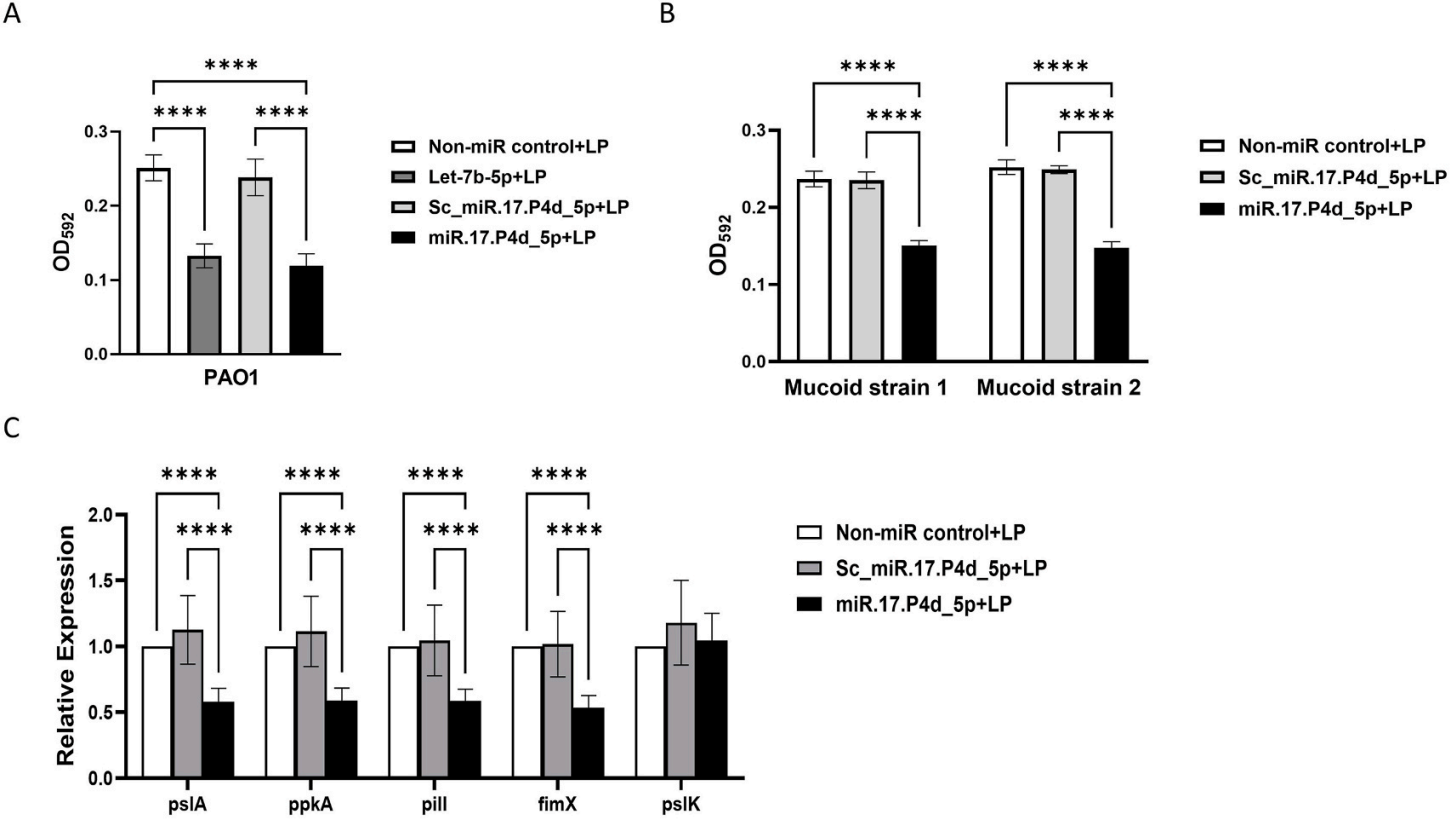
Effect of Td-miR.17.P4d_5p on biofilm formation and virulence gene expression in *P. aeruginosa*. Biofilm formation by PAO1 **(A)** and *P. aeruginosa* mucoid strains **(B)** after 48 h incubation in the absence (non-miR control) or presence of Td-Let-7b-5p, Td-Sc-miR.17.P4d_5p, or Td-miR.17.P4d_5p (1 μM). LP and cefotaxime were added to 5 × 10^7^ bacterial cells at T = 0 h in 200 μL MHB in 96-well plates and incubated at 37°C. Cefotaxime was used at 16 μg/mL (mucoid strain 1) or 32 μg/mL (PAO1 and mucoid strain 2). Td and LP were added at T = 24 h. After 48 h, biofilms were washed, stained with 200 μL 0.1% (w/v) crystal violet, and quantified by OD_592_. Analysis was performed using multiple comparison one-way ANOVA where results were significantly different when comparing treated group to control group with ****p < 0.00001, not significant (Not displayed, *p* > 0.01), n = 3 biological replicates. **(C)** Relative expression of *pslA, ppkA, pilI, fimX*, and *pslK* in PAO1 was quantified by qPCR after 48 h treatment with Td-Sc-miR.17.P4d_5p or Td-miR.17.P4d_5p (1 μM) in the presence of 32 μg/mL cefotaxime and LP, normalized to the housekeeping gene *proC*. Analysis was performed using multiple comparison two-way ANOVA where results were significantly different when comparing treated group to control group with ****p < 0.00001, not significant (Not displayed, *p* > 0.01), n = 3 biological replicates.

In the presence of LP and 32 μg/mL cefotaxime, delivery of Let-7b-5p via the DNAtd system, used as a positive control, significantly reduced PAO1 biofilm formation [13,14]. Notably, Td-miR.17.P4d_5p treatment also caused a significant reduction in biofilm formation compared with the scrambled control (Sc_miR.17.P4d_5p) (Fig. 6A). Furthermore, in the presence of LP and cefotaxime Td-miR.17.P4d_5p also significantly inhibited biofilm formation in two clinical mucoid *P. aeruginosa* strains: mucoid strain 1 at 16 μg/mL cefotaxime and mucoid strain 2 at 32 μg/mL cefotaxime (Fig. 6B).

Based on published studies and *in silico* target predictions, we investigated the molecular mechanisms by which Td-Let-7b-5p and Td-miR.17.P4d_5p inhibit PAO1 biofilm formation (Supplementary Table S4) [13]. qPCR analysis showed that, compared with the non-miR control, Td-Let-7b-5p significantly downregulated *hcp1* expression, while having no effect on the non-target gene *ppkA* (Supplementary Fig. S6). Td-miR.17.P4d_5p significantly reduced the expression of its predicted target genes *pslA, ppkA*, *pilI*, *and fimX* [[38], [39], [40], [41], [42]], without affecting the non-predicted gene *pslK* (Fig. 6C). Taken together, Td-miR.17.P4d_5p effectively suppresses biofilm formation in PAO1 and clinical mucoid strains by downregulating key biofilm-associated genes.

### 3.8 Direct binding validation of miR.17.P4d_5p to *P. aeruginosa* target genes by ssDNA hybridisation EMSA

To experimentally validate the predicted interactions between miR.17.P4d_5p and *P. aeruginosa* target genes, electrophoretic mobility shift assays (EMSAs) were performed using wild-type (WT) and mutant (Mut) DNA probes corresponding to the predicted binding regions. A mobility shift was interpreted as evidence of direct binding between miR.17.P4d_5p DNA oligo and the target probes, while loss of binding following mutation was used to assess interaction specificity.

**Fig. 7:**
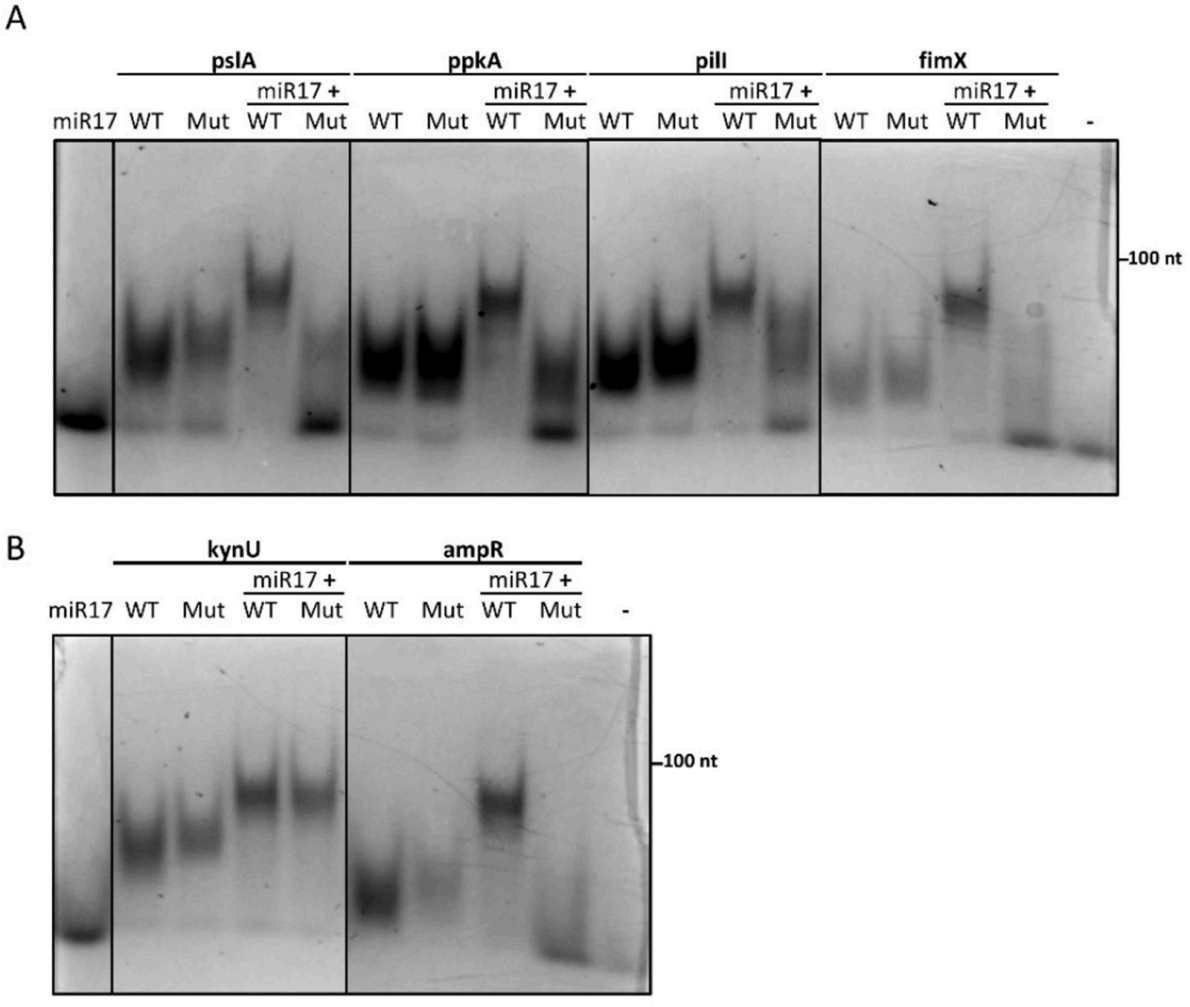
Direct binding validation of miR.17.P4d_5p to *P. aeruginosa* target genes by EMSA. **(A)** EMSA analysis of miR.17.P4d_5p DNA oligo binding to WT and mutant (Mut) DNA probes of biofilm formation genes *pslA, ppkA, pilI,* and *fimX*. The “−” lane contains 1× loading buffer alone and serves as a loading control, n = 3 biological replicates. **(B)** EMSA analysis of miR.17.P4d_5p DNA oligo binding to WT and mutant (Mut) DNA probes of *kynU* and *ampR*. The “−” lane contains 1× loading buffer alone and serves as a loading control, n = 3 biological replicates.

As shown in the results, EMSA analysis was conducted for four biofilm formation genes, *pslA, ppkA, pilI,* and *fimX*. Clear mobility shifts were observed when the miR.17.P4d_5p DNA oligo was incubated with the WT probes of all four genes, confirming direct interactions with the predicted target sequences. In contrast, mutation of the predicted binding sites abolished binding for the *pslA, ppkA,* and *fimX* probes, as evidenced by the absence of shifted complexes. For *pilI*, the mutant probe retained partial binding to miR.17.P4d_5p, suggesting that the introduced mutations only partially disrupted the interaction (Fig. 7A).

Further EMSA analysis was performed for the growth-associated gene *kynU* and the β-lactam resistance regulator *ampR*. Both the WT and Mut *kynU* probes exhibited substantial mobility shifts following incubation with miR.17.P4d_5p (Fig. 7B). Consistent with the *in silico* prediction results (Table 1), the calculated binding energy was only minimally affected by the introduced mutations, which likely explains the persistence of mutant probe-miR.17.P4d_5p DNA oligo complex formation. In contrast, a clear mobility shift was observed only for the WT *ampR* probe, whereas binding was not visible following mutation of the predicted target site (Fig. 7B). These findings indicate that the interaction between miR.17.P4d_5p and *ampR* is highly sequence-specific.

IntaRNA-based interaction models further support the EMSA findings by illustrating the predicted binding between miR.17.P4d_5p and the WT and mutant sequences of *pslA, pilI,* and *kynU*. These results recapitulate the observed binding behaviors in EMSA, showing complete loss of binding in the *pslA* mutant, partial retention of binding in the *pilI* mutant, and sustained binding in both WT and mutant *kynU* sequences (Supplementary Fig. S7). Together, these predictions are consistent with the EMSA results and further support the conclusion that miR.17.P4d_5p exhibits target-dependent binding specificity influenced by the integrity of the predicted interaction sites.

## 4 Discussion

Cystic fibrosis (CF) lung disease is sustained by a self-perpetuating cycle of impaired mucociliary clearance, chronic bacterial infection, excessive neutrophilic inflammation, and progressive structural lung damage [43,44]. Although CFTR modulator therapies have transformed clinical outcomes, persistent airway infection—particularly with *Pseudomonas aeruginosa*—and unresolved inflammation remain major causes of morbidity and mortality in individuals with CF [45,46]. Addressing this unmet therapeutic need, the present study demonstrates the dual antibacterial and anti-inflammatory properties of Td-miR.17.P4d_5p, a miRNA mimic delivered via a DNA tetrahedron (Td) nanostructure designed to simultaneously target bacterial virulence pathways and host inflammatory signaling [14,23].

We first synthesized and validated the structural integrity of Td-miR.17.P4d_5p, confirming the formation of a stable nanoscale tetrahedron capable of encapsulating miRNA-like DNA oligonucleotides without inducing cytotoxicity in CF bronchial epithelial cells. The construct exhibited efficient cellular uptake, supporting the suitability of Td-based nanostructures as delivery platforms for airway epithelium. We next confirmed and extended prior findings that miR-17-5p functions as a negative regulator of IL-8/CXCL8 expression in CF airway epithelial cells [12]. Delivery of Td-miR.17.P4d_5p markedly reduced IL-8 mRNA abundance as well as IL-8 protein production and secretion in CFBE and HBE cells stimulated with both laboratory and clinical *P. aeruginosa* culture filtrates. Given the central role of IL-8 in neutrophil recruitment and CF lung tissue injury [12,47,48], these results reinforce miR-17-5p, and a DNA mimic thereof, as a key endogenous suppressor of airway inflammation.

Crucially, this study also establishes that Td-miR.17.P4d_5p exerts direct antibacterial activity against *P. aeruginosa*. Treatment of PAO1 with Td-miR.17.P4d_5p significantly inhibited its growth, with increased enhanced antibacterial efficacy observed in the presence of the liposomal adjuvant LP2000. As was seen for *P. aeruginosa*, Td-miR.17.P4d_5p also interfered with *S. aureus* growth, albeit with differing effects at different timepoints in the three *S. aureus* strains. This indicates that the growth inhibitory effects of Td-miR.17.P4d_5p are not limited to *P. aeruginosa*, but similar to miR-101-3p, can impact on both Gram-negative and Gram-positive bacteria [14]. Furthermore, Td-miR.17.P4d_5p potentiated the bactericidal activity of cefotaxime against *P. aeruginosa* by reducing bacterial survival in both laboratory and mucus clinical isolates, likely through inhibition of the *ampR–ampC* β-lactam resistance pathway. The construct also robustly suppressed PAO1 biofilm formation—a hallmark of chronic CF infections—by downregulating key biofilm-associated genes, including *ppkA*, *pilI*, *fimX*, and *pslA* [[38], [39], [40], [41], [42]]. These findings highlight that miRNA-based therapeutics can selectively modulate bacterial virulence programmes rather than relying solely on traditional bactericidal pressure. Together, the results implicate Td-miR.17.P4d_5p as a promising nanotherapeutic agent with combined antibacterial and anti-inflammatory activity. This work positions Td-miRNA constructs within the emerging field of DNA-nanostructure-based antimicrobial therapeutics and underscores their translational potential for overcoming biofilm-associated antimicrobial resistance and addressing persistent infection and inflammation in CF.

DNA tetrahedrons (Tds) represent a rapidly advancing class of programmable nanomaterials distinguished by their precise self-assembly, structural rigidity, nuclease resistance, and excellent biocompatibility [22,49,50]. Since their development, Td architectures have been extensively explored as delivery vehicles for siRNAs, miRNAs, antisense oligonucleotides, and small-molecule therapeutics across models of cancer, inflammatory disease, and infection [[51], [52], [53], [54], [55], [56]]. In the setting of bacterial infection, Td-based delivery systems offer several notable advantages. Unlike conventional cationic transfection agents, Tds facilitate efficient oligonucleotide uptake without inducing cytotoxicity or compromising membrane integrity [49,57,58]. Prior studies using DNAtd-miR-101-3p demonstrated that Td-delivered miRNA mimics can enter both *Pseudomonas aeruginosa* and *Staphylococcus aureus*, suppress essential bacterial genes, impair biofilm formation, and potentiate β-lactam antibiotic activity [14]. Importantly, Td scaffolds alone exhibit no antibacterial activity, confirming that these effects are sequence-specific and directly mediated by the delivered miRNA. The present study builds on this foundation by showing that Td-miR.17.P4d_5p concurrently modulates host inflammatory IL-8 signaling and *P. aeruginosa* pathogenicity, reinforcing the suitability of Tds as multifunctional therapeutic platforms for complex inflammatory infections such as cystic fibrosis (CF).

Biofilm formation is a defining feature of chronic *P. aeruginosa* infection in CF airways. Biofilms shield bacteria from antibiotics, host immune responses, and environmental stressors, while promoting phenotypic heterogeneity and long-term persistence [[59], [60], [61]]. Within biofilms, bacterial tolerance to antibiotics can increase by up to a thousand-fold, rendering conventional antimicrobial approaches largely ineffective [62,63]. The challenge is further exacerbated by antimicrobial resistance (AMR). Recurrent and sustained antibiotic exposure in CF selects for resistant strains, including those with elevated β-lactamase expression, altered membrane permeability, and enhanced efflux systems [64,65]. Among these mechanisms, the *ampR–ampC* regulatory axis, targeted by Td-miR.17.P4d_5p, is a central determinant of β-lactam resistance in *P. aeruginosa* and a key contributor to treatment failure [33,66].

There is increasing recognition that effective management of chronic infections requires targeted *anti*-biofilm strategies. Rather than focusing solely on bacterial eradication, disrupting biofilm formation and maintenance can restore antibiotic susceptibility, reduce bacterial burden, and diminish inflammation-driven tissue injury [13,67]. Td-mediated miRNA delivery, exemplified by DNAtd-miR-101-3p and Td-miR.17.P4d_5p, represents a conceptual shift in the treatment of chronic infection and AMR. Whereas traditional antibiotics act by inhibiting growth or killing bacteria—thereby imposing strong selective pressures that favor the emergence of resistant mutants—miRNA-based strategies operate through a fundamentally different mechanism [14]. Instead of targeting bacterial viability, they selectively interfere with virulence pathways, regulatory networks of resistance, and biofilm-associated gene expression [24,[68], [69], [70]]. This multi-target, low–lethal-pressure regulatory model diminishes the evolutionary incentives driving resistance acquisition.

Crucially, this approach enables precise modulation of bacterial pathogenicity rather than survival. Bacterial traits essential for chronic infection—biofilm formation and resistance regulation—are substantially weakened but not lethally targeted. As a result, microbial community structure may remain relatively undisturbed, potentially minimizing the selective pressures that typically accelerate resistance evolution [13,14,71]. Such a strategy is particularly valuable in diseases like CF, where chronic, recurrent infections persist despite aggressive antibiotic therapy and where controlling pathogenic behaviors may be more biologically feasible and clinically impactful than complete bacterial eradication.

The major advantage of Td-miR.17.P4d_5p lies in its dual mechanism of action. By concurrently suppressing IL-8–mediated neutrophilic inflammation and directly inhibiting *P. aeruginosa* growth, biofilm formation, and antibiotic resistance, Td-miR.17.P4d_5p addresses two core drivers of CF lung disease. A second distinguishing feature is its mechanistic specificity. As shown by qPCR, and validated by EMSA using wild type and mutant target sequences, miR-17.P4d_5p selectively downregulates defined bacterial and host targets, thereby minimizing collateral effects on commensal microbiota and reducing the selective pressure that typically drives resistance evolution [13,69]. Third, the modular architecture of the Td platform enables rational optimization. Td size, charge, surface chemistry, and cargo composition can be systematically tuned to improve mucus penetration, bacterial entry, or epithelial cell targeting—an important consideration in CF airways characterized by viscous mucus and altered ionic conditions [72,73]. Finally, Td-miR.17.P4d_5p demonstrates antibiotic-sparing potential: by sensitizing bacteria to existing agents, it may reduce required antibiotic doses, limit treatment-related toxicity, and slow the emergence of antimicrobial resistance [3,64,65].

Despite these strengths, important limitations must be acknowledged which are beyond the scope of the current study. The current investigation is restricted to in vitro systems and cell lines rather than primary cells, uses DNA oligonucleotide mimics rather than ribonucleic acid-based miRNAs, and has limited translatability. The CF lung presents formidable delivery barriers—including dense mucus, abundant proteases, extracellular DNA, and a polymicrobial milieu—that may influence Td stability, penetration, and biodistribution in vivo [[74], [75], [76]]. Here only laboratory strains and non-CF isolates are used, and only cefotaxime is tested. Reporter assays and rescue experiments that would unequivocally validate the proposed mechanisms are lacking and should form the basis of future studies. Third, although miR-17-5p is an endogenous molecule, off-target effects by its DNA mimic in host cells remain a concern; comprehensive transcriptomic and proteomic analyses would be necessary to fully evaluate safety. Finally, challenges inherent to nucleic acid nanomedicine—such as scalable manufacturing, batch-to-batch reproducibility, and long-term physicochemical stability—would need to be carefully addressed before clinical translation.

Current CF therapies largely fall into three categories: CFTR modulators, antibiotics, and anti-inflammatory agents [44,45,48,[77], [78], [79], [80], [81], [82]]. While CFTR modulators have substantially improved outcomes for eligible individuals, they do not eradicate chronic infections or fully resolve established inflammation [48,[79], [80], [81],^83^]. Antibiotics remain central to managing airway infection but are increasingly limited by biofilms, AMR, and cumulative systemic toxicities [78]. Anti-inflammatory drugs—including corticosteroids and high-dose ibuprofen—carry significant side-effect burdens and do not target the underlying infectious stimulus [44,77]. Td-miR.17.P4d_5p has the potential to bridge these therapeutic gaps. By acting independently of CFTR genotype, it may benefit patients who cannot receive modulator therapy. Its targeted *anti*-biofilm and antibiotic-sensitizing effects directly address shortcomings of conventional antimicrobial strategies, while its selective suppression of IL-8 expression provides a more precise and potentially safer approach to controlling airway inflammation.

Several priorities will guide future translation. In vivo validation in CF animal models is essential to determine pharmacokinetics, biodistribution, mucus penetration, and immunogenicity. Development of aerosolized or inhalable Td formulations tailored to the biophysical properties of CF sputum represents a logical next step [76,84,85]. Combination studies with standard-of-care antibiotics and CFTR modulators will clarify how Td-miR.17.P4d_5p integrates into existing treatment regimens. Importantly, the broader implications of this work extend far beyond CF. Td-miR.17.P4d_5p′s *anti*-IL-8 effects were observed in a non-CF bronchial epithelial cell line infection model, thereby extending it's the potential clinical applications. Furthermore, demonstrating that host-derived miRNAs delivered via Td nanostructures can directly reprogram bacterial behavior introduces a new therapeutic paradigm for chronic infections and antimicrobial resistance.

In summary, this study establishes Td-miR.17.P4d_5p as a multifunctional nanotherapeutic capable of simultaneously suppressing airway inflammation and targeting key virulence and resistance pathways in *P. aeruginosa*. By uniting precise gene-level regulation with a biocompatible and tunable delivery platform, Td-miR.17.P4d_5p represents a promising step toward clinically translatable dual-action therapies for CF and other biofilm-driven infectious diseases.

## Ethics statement

No human or animal studies were carried out as part of this work.

## Data availability statement

All data available in the manuscript and as supplementary data. Further enquiries can be directed to the corresponding author.

## Funding sources statement

RCSI-Soochow StAR PhD Programme 2023.

## CRediT authorship contribution statement

**Yunfei Ye:** Conceptualization, Data curation, Formal analysis, Funding acquisition, Investigation, Methodology, Writing – original draft. **YinHeRichard Sun:** Data curation, Formal analysis, Investigation, Methodology, Writing – original draft. **Hawraa Shahrour:** Formal analysis, Methodology. **Irene Oglesby:** Supervision. **Yiran Zheng:** Funding acquisition, Supervision. **Catherine M. Greene:** Conceptualization, Funding acquisition, Project administration, Supervision, Writing – review & editing.

## Declaration of competing interest

The authors declare that they have no known competing financial interests or personal relationships that could have appeared to influence the work reported in this paper.
