# Supplementary material for: A DNA Tetrahedron–Delivered miR-17-5p mimic exerts dual anti-inflammatory and Anti-Pseudomonas aeruginosa effects in cystic fibrosis
Source: Noncoding RNA Res. 2026 Jul 29;20:72–85. doi: 10.1016/j.ncrna.2026.06.006 (PMC13451911; doi:10.1016/j.ncrna.2026.06.006)
Supplement: Supplementary file 1 [file mmc1.docx]

# Supplementary Figures

**Supplementary Fig. S1.** Nanosight measurement of concentration and size of Td-miR.17.P4d_5p oligo complex.

**Supplementary Fig. S2.** Cytotoxicity of 0.5 μM and 1 μM Td-miR.17.P4d_5p in CFBE and HBE cells was evaluated by LDH assay, n = 3 biological replicates.

**Supplementary Fig. S3.** Confocal microscopy imaging analysis of the uptake of Td-miR.17.P4d_5p mixed with LP by PAO1. PAO1 (1 × 10^6^ CFU/mL) was incubated with 1 μM 6-FAM-labeled Td-miR.17.P4d_5p + LP (0.04 μL LP per μL Td solution) for 4 h and analyzed by confocal microscopy. Scale bar represents 5 μm.

**Supplementary Fig. S4.** SH1000/ USA300/ BH1CC was grown in MHB with and without LP and Td-miR.17.P4d_5p for 24 h and plated at T = 0, 2, 4, 6, 8, and 24 h. 1 μM Td-miR.17.P4d_5p -/+ LP were added to 5 × 107 bacterial cells at T = 0 h. The number of viable cells was detected at different time points and presented as CFU/mL -/+ standard deviation. Analysis was performed using multiple comparison two-way ANOVA where results were significantly different when comparing treated group to control group with ** *p* < 0.001, * *p* < 0.01, not significant (Not displayed, *p* > 0.01), n = 3 biological replicates.

**Supplementary Fig. S5.** MIC values of cefotaxime in the presence or absence of 1μM Td-miR.17.P4d_5p, with bacterial growth assessed by OD600 measurements using a microplate reader (absorbance at 600 nm); sub-MIC concentrations were 8 μg/mL for PAO1 (Left) and 16 μg/mL for PA14 (Right). Analysis was performed using a Student’s t test where results were significantly different when comparing treated group to control group with **** *p* < 0.00001, ** *p* < 0.001, n = 3 biological replicates.

**Supplementary Fig. S6.** Relative expression of *hcp1* and *ppkA* in PAO1 was quantified by qPCR after 48 h treatment with Td-Let-7b-5p (1 μM) in the presence of 32 μg/mL cefotaxime and LP (0.04 μL LP per μL Td solution), normalized to the housekeeping gene *proC*. Analysis was performed using multiple comparison two-way ANOVA where results were significantly different when comparing treated group to control group with **** *p* < 0.00001, not significant (Not displayed, *p* > 0.01), n = 3 biological replicates.

**Supplementary Fig. S7.** IntaRNA visualization of base-pairing interactions between miR.17.P4d_5p and WT and mutant (Mut) RNA sequences of *pslA, pilI*, and *kynU*. Top strand indicates gene of interest, bottom strand indicates miR.17.P4d_5p.
